# Supplementary material for: Real-World Comparison of Transcatheter Versus Surgical Aortic Valve Replacement in the Era of Current-Generation Devices
Source: J Clin Med. 2023 Jan 10;12(2):571. doi: 10.3390/jcm12020571 (PMC9864945; doi:10.3390/jcm12020571)
Supplement: Supplementary file 1 [file jcm-12-00571-s001.zip › jcm-2129850-supplementary.pdf]

Table S1. Claims and Disease Codes Used in Patients Selection and Analysis

Treatment / Disease	Codes
SAVR	O1793, O1793010, O1793050, O1793080, O1793200, O1793210, O1793250, O1793280
TAVR	M6580, M6580010, M6580050, M6581, M6581010, M6581050, M6582, M6582020, M6582050
Infective Endocarditis	A52.0, A54.8, B37.6, I33, I38, I39.8
Aortic Regurgitation	I35.1
Permanent Pacemaker Insertion	O0203, O0203010, O0203020, O0203030, O0203040, O0203050, O0204, O0204010, O0204020, O0204030, O0204040, O0204050
Ischemic Stroke	I63
Intracranial Hemorrhage	I60, I61, I62
Hypertension	I10, I11, I12, I13, I14, I15
Diabetes	E10, E11, E12, E13, E14
Heart Failure	I50
CAD	I20
PCI	M6551, M6552, M6571, M6572, M6561, M6562, M6563, M6564
Dyslipidemia	E78
Aortic Disease	I71, I72
PAD	I73
AF	I48

COPD	J44
CKD	N18
ESRD	N185

COPD: chronic obstructive pulmonary disease; PCI: percutaneous coronary intervention; PAD: peripheral artery disease; AF: atrial fibrillation; CKD: chronic kidney disease; ESRD: end-stage renal disease; SAVR: surgical aortic valve replacement; TAVR: transcatheter aortic valve replacement

Table S2. Baseline Patient Characteristics before 1:1 Propensity Matching

	**TAVR N=1,269**	**SAVR N=3,354**	**p-value**
**Age (mean ± SD)**	80.2 ± 5.4	74.4 ± 5.3	< 0.001
**Age (median, IQR)**	80, 7	74, 8	< 0.001
**Age categories**			< 0.001
**65 ~ 79**	568 (44.8)	2,746 (81.9)	
**≥ 80**	701 (55.2)	608 (18.1)	
**Female**	669 (52.7)	1,675 (49.9)	0.092
**Hypertension**	1,190 (93.8)	2,923 (87.1)	< 0.001
**Diabetes**	881 (69.4)	2,124 (63.3)	< 0.001
**COPD**	282 (22.2)	551 (16.4)	< 0.001
**CAD**	809 (63.8)	1,841 (54.9)	< 0.001

**Previous PCI**	150 (11.8)	110 (3.3)	< 0.001
**Dyslipidemia**	1,170 (92.2)	2,907 (86.7)	< 0.001
**Heart failure**	723 (57.0)	1,619 (48.3)	< 0.001
**AF**	229 (18.0)	657 (19.6)	0.234
**Previous stroke**	283 (22.3)	520 (15.5)	< 0.001
**Aortic disease**	57 (4.5)	243 (7.2)	0.001
**PAD**	406 (32.0)	880 (26.2)	< 0.001
**CKD**	174 (13.7)	281 (8.4)	< 0.001
**ESRD**	59 (4.6)	104 (3.1)	0.011

Values are mean plus–minus ± standard deviation or number (%)

COPD: chronic obstructive pulmonary disease; PCI: percutaneous coronary intervention; PAD: peripheral artery disease; AF: atrial fibrillation; CKD: chronic kidney disease; ESRD: end-stage renal disease; SAVR: surgical aortic valve replacement; TAVR: transcatheter aortic valve replacement

Table S3. Antithrombotic Medication

*	TAVR	SAVR
**Anticoagulation alone**	7 (0.7)	69 (6.5)
**Anticoagulation and antiplatelet**	79 (7.5)	253 (23.9)
**Antiplatelet alone**	739 (69.7)	163 (15.4)
**Dual antiplatelet**	231 (21.8)	566 (53.4)

**No antithrombotics**	4 (0.4)	9 (0.8)

TAVR: transcatheter aortic valve replacement; SAVR: surgical aortic valve replacement

* P-value < 0.001

Table S4. Valves used in procedures

A : TAVR group

Valve	Case Number
**Edwards Sapien 3**	612
**CoreValve Evolut R**	387
**LOTUS**	26
**Missing Data**	35

B : SAVR group

Valve	Case Number	Valve Material
**Carpentier Edwards Perimount Magna**	553	Tissue
**Trifecta**	292	Tissue
**Mitroflow Aortic Pericardial Heart Valve**	84	Tissue
**Hancock II**	81	Tissue
**Epic Supra**	19	Tissue
**SJM Epic**	13	Tissue
**Mosaic**	10	Tissue

**Avalus**	5	Tissue
**Pericarbon More Pericardial**	2	Tissue
**Soprano**	1	Tissue

Table S5. Concomitant Procedures (TAVR & SAVR)

**TAVR**	**Case (%)**
**PCI**	11.7
**Balloon angioplasty**	1.1
**Rotational atherectomy**	0.1

**SAVR**	**Case (%)**
**CABG**	17.5%
**MVR**	6.4%
**MV repair**	3.8%
**TVR**	0.1%
**TV repair**	4.8%
**Ascending aorta replacement**	11.2%
**Aortic arch replacement**	5.4%
**Descending aorta replacement**	0.1%
**ASD closure**	1.3%
**myectomy**	2.1%

Table S6. Approach of TAVR

TAVR	Case (%)
**Femoral**	97.8%
**Ascending aorta**	0.9%
**Apical**	1.2%

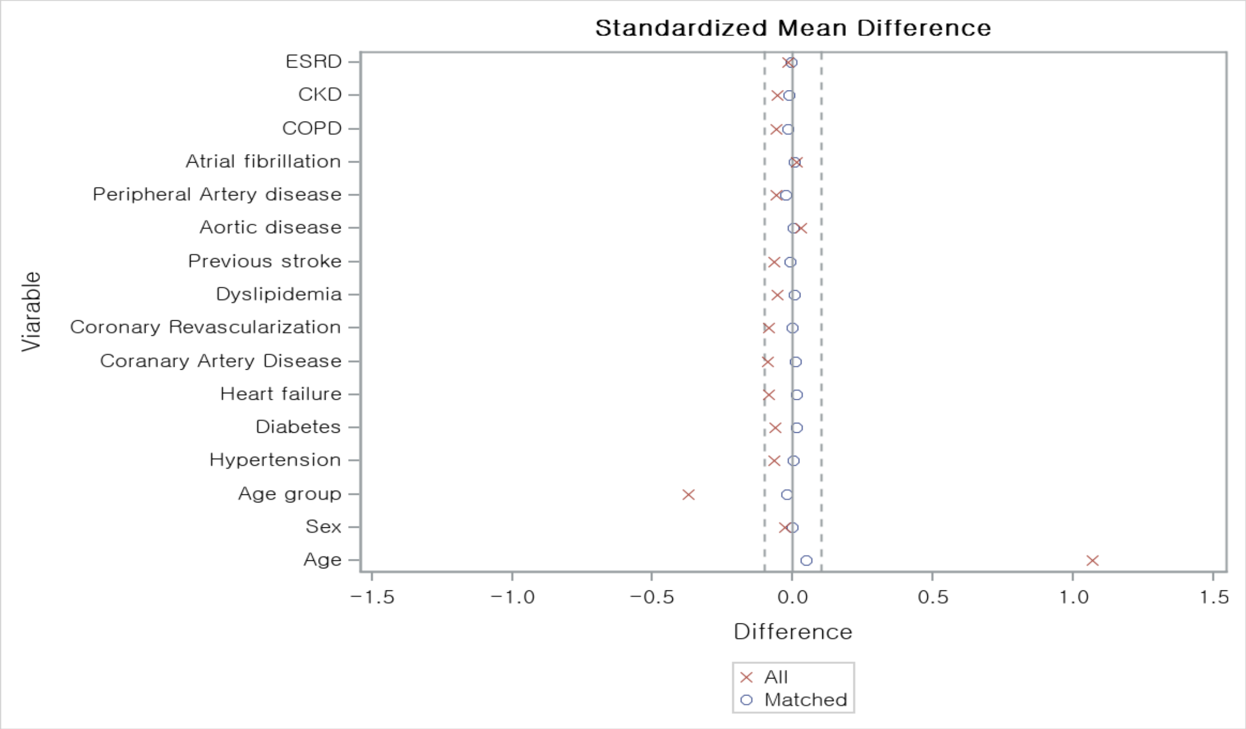

Figure S1. Love plot for Standardized mean difference before and and after propensity matching
